# Safety of nursing professionals and patient facing COVID-19 pandemic in critical care unit

**DOI:** 10.1590/1518-8345.6317.3861

**Published:** 2023-03-27

**Authors:** Juliana Rizzo Gnatta, Rita de Cássia Almeida Vieira, Luciana Soares Costa Santos, Sirlene Luz Penha, Giovanna Nogueira Sanchez, Jussiely Cunha Oliveira, Eduesley Santana-Santos, Maria de Fatima Fernandes Vattimo

**Affiliations:** 1 Universidade de São Paulo, Escola de Enfermagem, São Paulo, SP, Brazil.; 2 Scholarship holder at the Coordenação de Aperfeiçoamento de Pessoal de Nível Superior (CAPES), Brazil.; 3 Universidade Federal de Sergipe, Aracaju, SE, Brazil.

**Keywords:** COVID-19, Risk Management, Occupational Health, Patient Safety, Personal Protective Equipment, Infection Control, COVID-19, Gestão de Riscos, Saúde do Trabalhador, Segurança do Paciente, Equipamento de Proteção Individual, Controle de Infecção, COVID-19, Gestión de Riesgos, Salud Laboral, Seguridad del Paciente, Equipo de Protección Personal, Control de Infecciones

## Abstract

**Objective:**

to evaluate nursing professionals and patient safety culture during the professional performance in the care of suspected or infected patients with COVID-19.

**Method:**

a cross-sectional study carried out with 90 professionals from critical care units of two teaching hospitals. An instrument for sociodemographic characterization and health conditions was used, in addition to the constructs “Nursing professional and patient safety” and the Hospital Survey on Patient Safety Culture. Univariate analyzes were performed between the diagnosis of COVID-19 and the characteristics of Nursing professionals, applying Kendell’s correlation between the constructs.

**Results:**

the COVID-19 diagnosis presented a significant statistical difference between nursing professionals that worked for more than six years at the critical care unit (p=0.020) and the items of the construct “Nursing professional and patient safety” regarding the doubts about how to remove the personal protective equipment (p=0.013) and safety flow (p=0,021). The dimensions 2 (p=0.003), 3 (p=0.009), 4 (p=0.013), 6 (p<0.001), and 9 (p=0.024) of the Hospital Survey on Patient Safety Culture were associated with the accomplishment of training.

**Conclusion:**

a higher professional nursing experience time was associated with non-infection by COVID-19. The perception of the safety culture of the patient was related to the accomplishment of training.

## Resumo

**Objetivo:**

avaliar a segurança dos profissionais de enfermagem e a cultura de segurança do paciente durante a atuação profissional no atendimento de pacientes suspeitos ou com COVID-19.

**Método:**

estudo transversal realizado com 90 profissionais de unidades críticas de dois hospitais-escola. Foi utilizado um instrumento para caracterização sociodemográfica e condições de saúde, além dos constructos “Segurança do profissional e do paciente” e *Hospital Survey on Patient Safety Culture*. Foram realizadas análises univariadas entre o diagnóstico de COVID-19 e as características dos profissionais, aplicando-se a correlação de Kendell entre os constructos.

**Resultados:**

o diagnóstico de COVID-19 apresentou diferença estatisticamente significativa entre os profissionais que trabalhavam há mais de seis anos na unidade (p=0,020) e os itens do constructo “Segurança do profissional e do paciente” referentes às dúvidas sobre como retirar o equipamento de proteção individual (p=0,013) e fluxo seguro (p=0,021). As dimensões 2 (p=0,003), 3 (p=0,009), 4 (p=0,013), 6 (p<0,001) e 9 (p=0,024) do *Hospital Survey on Patient Safety Culture* foram associadas à realização de treinamentos.

**Conclusão:**

maior tempo de experiência profissional do profissional de enfermagem esteve associado a não infecção por COVID-19. A percepção de cultura de segurança do paciente esteve relacionada à realização de treinamentos.

## Resumen

**Objetivo:**

evaluar la seguridad de los profesionales de enfermería y la cultura de seguridad del paciente durante su actuación profesional en el cuidado de pacientes sospechosos o con COVID-19.

**Método:**

estudio transversal realizado con 90 profesionales de unidades críticas de dos hospitales universitarios. Se utilizó un instrumento de caracterización sociodemográfica y de condiciones de salud, además de los constructos “Seguridad del profesional y del paciente” y *Hospital Survey on Patient Safety Culture*. Se realizaron análisis univariados entre el diagnóstico de COVID-19 y las características de los profesionales y se aplicó la correlación de Kendell entre los constructos.

**Resultados:**

el diagnóstico de COVID-19 mostró diferencia estadísticamente significativa entre los profesionales que actuaron por más de seis años en la unidad (p=0,020) y los ítems del constructo “Seguridad del profesional y del paciente” referentes a dudas sobre cómo retirar el Equipo de Protección Personal (p=0,013) y flujo seguro (p=0,021). Las dimensiones 2 (p=0,003), 3 (p=0,009), 4 (p=0,013), 6 (p<0,001) y 9 (p=0,024) del *Hospital Survey on Patient Safety Culture* se asociaron a la realización de formación.

**Conclusión:**

mayor tiempo de experiencia profesional de enfermería se asoció con la no infección por COVID-19. La percepción de la cultura de seguridad del paciente se relacionó con la realización de entrenamiento.

**Highlights**

(1) The management performs a fundamental role in patients and occupational safety.

(2) The COVID-19 diagnosis was associated with the “Overall perceptions of patient safety”.

(3) The training and support of the management team were essential to the perception of safety.

## Introduction

Experiencing a pandemic has several negative consequences, especially for nursing, a workforce that is at the forefront of hospital care. For these professionals, the development of new functions during the pandemic adds to the responsibilities of comprehensive care of managers and team leaders, in decision-making management, forecasting and provision of equipment and materials, implementing control and prevention strategies in health care, among others. These attributions were decisive for the nursing team, whose responsibility is to value their own safety and that of the patient as one of the fundamental pillars of quality in the provision of safe care^1^ ^-^ ^3^. 

The pressure of the pandemic scenario, many times chaotic and stressful, makes the nursing professional develop resilience in managing a new organization of the work process and assistance flow, besides adapting the reality of the care to the lack and restriction of equipment, beds, inputs, especially the Personal Protective Equipment (PPE). Based on that new scenario, a restructuring of assistance flows was carried out in hospitals to reduce the dissemination of the disease, as the recognition of the factors that interfere with the quality of the care, compromise the patient’s safety and cause adverse events. To guarantee the safety of professionals and patients it was necessary to change the patient safety culture and the educational process of the health team, with the continuous improvement of good safety and communication practices^3^ ^-^ ^6^.

During the COVID-19 pandemic, nursing was exposed to: work overload, high virus transmissibility, manipulation of specific protective equipment and high technology, which led health team members to physical and mental exhaustion in the workplace experienced worldwide^7^. Thus, the attitudes of nursing professionals, their training, the availability and safe use of PPE, psychological follow-up, peer support and workload influence workers in this area regarding the assessment of the safety of patients who were hospitalized. during the COVID-19 pandemic^8^.

In this context, the present study carried out in two teaching hospitals in different Brazilian states, had as objective to evaluate nursing professionals and patient safety culture during the professional performance in the care of suspected or infected patients with COVID-19.

## Method

### Study design

This is an exploratory cross-sectional study carried out in critical care units (prompt care, Intensive Care Unit (ICU)/semi-intensive and surgical center), guided by the STROBE (Strengthening the Reporting of Observational Studies in Epidemiology)^9^ tool. 

### Setting and sample size of the study

We carried out the study in two teaching hospitals, one being a University Hospital located in São Paulo city SP, Brazil and the other in Lagarto city/Sergipe (SE), Brazil, both a secondary level and with open doors for urgent care and clinic, pediatric, general surgery and orthopedic emergency. We justify the choice to evaluate the safety of nursing professional and patient safety culture through, respectively, two constructs, “Professional nursing and patient safety” and the “Patient safety culture” in two teaching hospitals because, although both have different times of operation and consolidation phases of the work process, in the COVID-19 pandemic both hospitals needed a restructure of the working process and assistance flow beside the continuous care improvement conduction. The Research Ethics Committee approved the research in its proper institutional instances under the Certificate of Presentation for Ethical Appreciation (in Portuguese *Certificado de Apresentação de Apreciação Ética*-CAAE) No. 31543420.0.3001.0076 opinion no 4.159.508 and 4.194.119. 

We estimated the intentional sampling with 90 nursing professionals that were operating in the direct care of the suspected or confirmed patient infection by COVID-19, that worked for at least a month in the sector and that accepted to participate in the study: 47 professionals from São Paulo’s hospital (20 nurses and 27 nurse technicians), and 43 from Sergipe’s hospital (18 nurses and 25 technicians). We excluded professionals in management positions that were on vacation or had medical licenses during the period of data collection. In the three months (October 1^st^ to December 30^th^, 2020). We carried out daily visits in both research units for the inclusion of participants that met the study eligibility criteria and accepted participation through the signing of the Informed Consent Form (ICF). During the daily visitations, the research team utilized the recommended PPE according to the unit’s institutional protocol where we conducted the data collection. The nursing professionals responded to the instrument with sociodemographic and health condition data to the previously validated constructs “Professional and patient safety” and Hospital Survey on Patient Safety Culture (HSOPSC)^10^.

For the security evaluation of the nursing professionals in operation during the care of suspected patients or with COVID-19, we applied a construct entitled “Professional nursing and patient safety.” The questionnaire consists of 17 items in the Likert type scale, with five measuring points: 1. Strongly disagree, 2. Disagree, 3. Neither agree nor disagree, 4. Agree, 5. Strongly agree, aiming to attribute a numerical value to the given answers by the participants according to the level of agreement with the statements. 

We elaborated the construct “Professional and patient safety” and we submitted to face and content validity in 2020. For that, a committee composed of three nurses specialized in nursing management, patient security and infection control and prevention related to the health care thematic evaluated the construct for validation. We invited the specialists to participate in the validation through an invitation letter sent via email with the ICF. They analyzed the instrument items regarding the content (relevance of the items in the instrument) and adequacy to measure with clarity what needs it proposes to measure (face validity). It was considered the level of agreement of 80% among the judges for each item to be evaluated^11^. We carried out the validation phase of the construct at the Escola de Enfermagem at Universidade de São Paulo (USP).

Furthermore, we evaluated the patient security culture in the units through the Hospital Survey on Patient Safety Culture (HSOPSC) instrument. The HSOPSC was validated in Brazil it contains 42 items distributed in 12 dimensions: “Teamwork within units” (Dimension 1 - composed of three items), “supervisor/manager expectations and actions promoting patient safety” (Dimension 2 - composed of four items), “organizational learning - continuous improvement” (Dimension 3 - composed of three items), “Management support for patient safety” (Dimension 4 - composed of three items), “Overall perceptions of patient safety” (Dimension 5 - composed of four items), “Feedback and communication about error” (Dimension 6 - composed of three items), “Communication openness” (Dimension 7 - composed of three items ), “Frequency of events reported” (Dimension 8 - composed by three items), “Teamwork across units” (Dimension 9 - composed of four items), “Staffing” (Dimension 10 - composed of four items), “Handoffs & transitions” (Dimension 11 - composed of four items), “Non-punitive response to error” (Dimension 12 - composed of three items). Scores over 75 suggest strengthened areas regarding patient security and scores under 50 indicate fragile areas. For the data analysis concerning the HSOPSC, the 16 reverse items (all items from dimensions 11 and 12, three items from dimension 10, two items from dimensions 2 and 5 and one item from dimensions 4 and 7 were inverted and we analyzed the percentage of positive answers applying the formula: % of positive answers from dimension X = [number of positive answers from dimension X/total number of valid answers to the dimension X (we will exclude positive, neutral, and negative from missing data)] x 100^10^. 

We collected sociodemographic data and COVID-19 data from nursing professionals. The categorical variables analyzed were: sex (male and female), professional category (nurse technicians and nurses), work shift (morning, afternoon, evening), place of work (prompt care, surgical center, ICU/semi-intensive, intensive therapy), municipality of residence, health conditions, COVID-19 diagnosis (yes, no), coagulopathy and/or septic shock (yes, no) during hospitalization and the quantitative variables: age (years), time experience in the care unit (years), time (days) and frequency of sick leave by COVID-19 (days). 

### Data analysis

We carried out the analysis of the sociodemographic data and COVID-19 infection through descriptive statistics using the R 4.1.1 software. We carried out descriptive statistics for every variable aiming at the overall characterization of the sample. We described the qualitative variables through absolute and relative frequency. For the continuous and discrete numerical variables, we calculated the average, standard deviation, median, and variation. We applied the Cronbach alpha coefficient to the construct “Professional and patient safety” and to the HSOPSC instrument to verify the data reliability and intern consistency, establishing as evidence of satisfactory internal consistency values superior to 0,70. 

The association of COVID-19 infection between variables: sociodemographic characteristics, professionals and the construct “Professional and patient safety” utilized Pearson’s chi-squared test or Fisher’s exact test. In the identification of the correlation between the HSOPSC dimensions and the “Professional and patient safety”, we applied the Kendall correlation coefficient.

We considered α de 5% for all analysis.

## Results

We included 90 nursing professionals (47 professionals from São Paulo and 43 from Sergipe) that answered the data collection instruments and attended the research eligibility criteria. Among participants, most were female (77.78%), with an average age of 39.4 years old, most were nursing technicians (56.67%) that worked at the prompt care unit (47.78%), in the morning shift (62.22%). Most professionals worked less than five years in the service (60.00%) and in the care units where they were located (84.44%), with an average professional experience of 8-10 years (Table 1).

**Table 1:** Nursing professional characterization (n=90). São Paulo, SP; Lagarto, SE, Brazil, 2020

Variable	n	%
*Sex*		
Female	70	77.78
Male	20	22.22
Age, mean/SD (years)	39.4/9.11	
*Professional category*		
Nursing technicians	51	56.67
Nurses	39	43.33
Place of work		
Prompt care	43	47.78
Surgical center	28	31.11
Intensive Care Unit	19	21.11
Work shift		
Morning	56	62.22
Evening	34	37.78
Working time in the hospital, mean/SD (years)	2.76/1.73	
*Experience time in the hospital*		
< 1 year	29	32.22
1 to 5 years	25	27.78
> 5 years	36	40.00
Working time in the located unit, mean/SD (years)	2.02/0.86	
*Experience time in the located care unit*		
< 1 ano	18	20.00
1 a 5 anos	58	64.44
> 5 anos	14	15.56
Experience time of the professionals, mean/SD (years)	8.10 (9.75)	

Regarding the professionals’ COVID-19 infection, 41.11% were positive, presenting various symptoms such as: mental confusion (97.30%), dizziness (81.08%), dyspnea (78.38%), chest pain (78.38%), diarrhea (64.87%), fever (59.46%), cough (59.46%), myalgia (59.46%), fatigue (45.95%), hyposmia (43.24%), anosmia (37.84%) and headache (21.62%). Regarding the time of sick leave of those infected by COVID-19, 91.89% (34 professionals) were isolated from work for 12.72 days, on average (SD=3.87); however, none of them needed hospitalization. 

We observed statistical significance when analyzing the working time in the care unit and infection by COVID-19 (p=0,020), being more frequent the infection on the professionals that worked more than six years than those with less working time in the unit.

In the evaluation of “Professional nursing and patient safety,” we verified that 97.65% of nursing professionals did the training for the utilization of PPE, 81.93% did the training for hand disinfection and 76.74% did the training for COVID-19 prevention. In Table 2 we observe that comparing the “Professional nursing and patient safety” instrument with the COVID-19 diagnosis among nursing professionals, we evidenced statistically significant differences only between the items doubts on removing the PPE (p=0.013) and safe patient flow (p=0.021) with COVID-19 infection. 

In the evaluations of the HSOPSC dimensions, we observed scores over 75 in domain 1 for COVID-19 infection and conduction of COVID-19 training. We observed the lowest scores in dimensions 10 and 11 (Table 3). There was a statistically significant difference between the presence of COVID-19 and dimension 5 (p=0.038). When analyzing the conduction of COVID-19 training for professionals with HSOPSC dimensions, we observed a statistically significant difference with dimension 2 (p=0.003), dimension 3 (p=0.009), dimension 4 (p=0.013), dimension 6 (p<0.001) and dimension 9 (p=0.024).

**Table 2:** Comparison between the items of the validated construct “Professional Nursing and patient safety” and the COVID-19 diagnosis of nursing professionals (n=90) from both teaching hospitals. São Paulo, SP; Lagarto, SE, Brazil, 2020

PROFESSIONAL AND PATIENT SAFETY	COVID-19 DIAGNOSIS						p-value*
	YES n (%)			NO n (%)			
	Disagree or Strongly disagree	Indifferent	Agree or Strongly agree	Disagree or Strongly disagree	Indifferent	Agree or Strongly agree	
1. I consider that my knowledge about the use of PPE^†^ is sufficient to care for patients with suspected or infected COVID-19	1 (2.7)	2 (5.4)	34 (91.9)	4 (7.5)	1 (1.9)	48 (90.6)	0.411
2. I have questions about how to correctly put on the PPE^†^	31 (83.8)	-	6 (16.2)	48 (90.6)	-	5 (9.4)	0.311
3. I have questions about how to safely remove the PPE^†^	28 (75.7)	1 (2.7)	8 (21.6)	46 (86.8)	3 (5.7)	4 (7.5)	0.013
4. I have questions about which PPE I should use to assist suspected or infected patients with COVID-19	32 (86.5)	1 (2.7)	4 (10.8)	50 (94.3)	1 (1.9)	2 (3.8)	0.282
5. In my unit^‡^ there are visible instructions about the correct use of PPE^†^	18 (48.7)	2 (5.4)	17 (45.9)	18 (34.0)	1 (1.9)	34 (64.1)	0.131
6. There was a restriction on the amount of PPE^†^ available for use	11 (29.7)	2 (5.4)	24 (64.9)	20 (37.7)	1 (1.9)	32 (60.4)	0.769
7. I know how to conduct the hand disinfection technique with water and soap properly	-	1 (2.7)	36 (97.3)	-	1 (1.9)	52 (98.1)	0.430
8. I know how to conduct the hand disinfection technique with alcoholic products properly	-	1 (2.7)	36 (97.3)	-	-	53 (100.0)	0.574
9. I believe that hand disinfection is important for the prevention of COVID-19 infection	-	1 (2.7)	36 (97.3)	1 (1.9)	-	52 (98.1)	0.747
10. The patient care flow in the unit where I work^‡^ was modified after the beginning of the pandemic	4 (10.8)	2 (5.4)	31 (83.4)	4 (7.5)	2 (3.8)	47 (88.7)	0.865
11. I received training about flow care of suspected patients or infected by COVID-19 in the unit where I work^‡^	8 (21.6)	2 (5.4)	27 (73.0)	11 (20.7)	-	42 (79.3)	0.481
12. I consider the care flow of the unit where I work safe for the suspected patients or infected by COVID-19	17 (46.0)	6 (16.2)	14 (37.8)	11 (20.8)	5 (9.4)	37 (69.8)	0.021
13. I consider the care flow in the unit I work‡ safe for patients that do not present suspect or infection by COVID-19	20 (54.1)	4 (10.8)	13 (35.1)	21 (39.6)	3 (5.7)	29 (54.7)	0.421
14. I believe that the care flow in the unit I work^‡^ could improve	6 (16,2)	5 (13.5)	26 (70.3)	8 (15.1)	4 (7.5)	41 (77.4)	0.903
15. I believe that the actions carried out by the manager of my unit^‡^ support and promote the security of professionals	5 (13.5)	9 (24.3)	23 (62.2)	9 (17.0)	6 (11.3)	38 (71.7)	0.476
16. I believe that the actions carried out by the manager of my unit^‡^ support and promote the security of patients	4 (10.8)	8 (21.6)	25 (67.6)	5 (9.4)	5 (9.4)	43 (81.2)	0.203

*Pearson’s chi-squared test or Fisher’s exact test. ^†^PPE = Personal protective equipment; ^‡^Consider the longer operating time unit

**Table 3:** HSOPSC^*^ Score by overall dimension and professionals’ infection (n=90) by COVID-19 and COVID-19 training. São Paulo, SP; Lagarto, SE, Brazil, 2020

HSOPSC scale^*^	% of positive answers					
	COVID-19		p-value^†^	COVID-19 training		p-value^†^
	NO	YES		NO	YES	
Dimension 1	76.10	75.68	0.870	68.33	80.30	0.182
Dimension 2	57.55	59.03	0.743	43.75	61.92	0.003
Dimension 3	53.77	52.78	0.798	36.25	58.85	0.009
Dimension 4	44.97	46.30	0.764	31.67	49.49	0.013
Dimension 5	40.41	50.23	0.038	51.25	41.54	0.102
Dimension 6	61.64	51.85	0.186	28.33	67.18	<0.001
Dimension 7	53.46	45.37	0.217	48.33	51.28	0.485
Dimension 8	35.85	37.96	0.785	28.33	39.49	0.128
Dimension 9	44.03	33.56	0.078	27.50	44.10	0.024
Dimension 10	24.06	25.68	0.825	22.50	25.00	0.854
Dimension 11	18.40	27.78	0.152	18.75	22.31	0.862
Dimension 12	47.14	48.15	0.872	51.67	46.15	0.568

*HSOPSC = Hospital Survey on Patient Culture*;* ^†^Wilcoxon-Mann-Whitney test

Table 4 data demonstrate the significant statistical correlation between workload, working time and actions carried out by managers (questions 11 to 16 of the “Professional nursing and patient safety” instrument) in front of the COVID-19 pandemic and HSOPSC dimensions. The results reveal serious or weak correlations between the HSOPSC dimensions and the analyzed variables. We observed higher positive correlations between domains 2 (τ = 0.40, p< 0.001), 3 (τ = 0.38, p< 0.001), 6 (τ = 0.38, p< 0.001) and 9 (τ = 0.37, p< 0.001), with an evaluation of professionals nursing regarding the patient security in their work unit at the hospital. Conversely, the flow of service in the work unit for patients with COVID-19 and the actions carried out by managers for the safety of professionals presented a negative correlation with domain 12 “Non-punitive response to error”.

**Table 4:** Correlation between the HSOPSC^*^ dimensions and the actions carried out by managers (questions 11 to 16 of the “Professional and patient safety”), workload and working time of professionals (n=90). São Paulo, SP; Lagarto, SE, Brazil, 2020

Variables	HSOPSC* SCALE DIMENSIONS - Correlation (p^†^-value)											
	1	2	3	4	5	6	7	8	9	10	11	12
Weekly workload	-0.09	0.01	-0.13	-0.18	0.08	-0,25	-0.24	-0.10	-0.24	0.02	0.19	0.14
	(0.386)	(0.904)	(0.155)	(0.069)	(0.427)	(0.010)	(0.018)	(0.316)	(0.010)	(0.816)	(0.058)	(0.142)
Working time in the hospital	-0.30	-0.11	-0.27	-0.12	-0.03	-0.15	-0.17	0.08	-0.15	-0.07	0.12	0.06
	(<0.001)	(0.220)	(0.002)	(0.183)	(0.763)	(0.089)	(0.070)	(0.395)	(0.084)	(0.434)	(0.180)	(0.478)
Working time in the unit	-0.06	-0.09	-0.11	-0.10	-0.02	-0.24	-0.22	0.06	-0.20	-0.06	0.29	-0.02
	(0.562)	(0.349)	(0.226)	(0.277)	(0.796)	(0.011)	(0.025)	(0.495)	(0.031)	(0.547)	(0.003)	(0.815)
Patient safety	0.23	0.40	0.38	0.29	-0.08	0.38	0.10	0.28	0.37	0.05	-0.18	-0.24
	(0.023)	(<0.001)	(<0.001)	(0.003)	(0.428)	(<0.001)	(0.300)	(0.004)	(<0.001)	(0.579)	(0.070)	(0.013)
Patient flow training	-0.02	0.34	0.09	0.09	-0.10	0.19	0.11	0.20	0.06	0.05	-0.05	-0.20
	(0.862)	(<0.001)	(0.311)	(0.308)	(0.292)	(0.043)	(0.231)	(0.033)	(0.515)	(0.579)	(0.626)	(0.028)
Safe flow of COVID-19 patient	0.07	0.24	0.14	0.16	-0.08	0.20	0.12	0.22	0.33	-0.09	-0.19	-0.39
	(0.444)	(0.009)	(0.096)	(0.070)	(0.377)	(0.025)	(0.174)	(0.015)	(<0.001)	(0.299)	(0.036)	(<0.001)
Safe flow of non-COVID-19 patient	-0.02	0.15	0.05	0.18	-0.06	0.13	-0.01	0.26	0.18	-0.01	-0.10	-0.22
	(0.819)	(0.102)	(0.535)	(0.041)	(0.513)	(0.139)	(0.896)	(0.005)	(0.038)	(0.907)	(0.260)	(0.015)
Improved flow	-0.19	-0.22	-0.14	-0.14	0.16	-0.28	-0.03	-0.25	-0.23	0.05	0.21	0.24
	(0,038)	(0.017)	-108	(0.135)	(0.091)	(0.002)	(0.742)	(0.007)	(0.010)	(0.605)	(0.025)	(0.009)
Actions of managers for professionals safety	0.10	0.27	0.20	0.09	-0.05	0.22	0.04	0.05	0.17	-0.02	-0.14	-0,39
	(0.271)	(0.004)	(0.024)	(0.299)	(0.577)	(0.014)	(0.679)	(0.610)	(0.054)	(0.814)	(0.128)	(<0.001)
Actions of managers in patient safety	0.08	0.29	0.26	0.14	-0.16	0.23	0.04	0.11	0.23	0.04	-0.17	-0.16
	(0.384)	(0.002)	(0.004)	(0.137)	(0.089)	(0.013)	(0.665)	(0.221)	(0.010)	(0.690)	(0.071)	(0.072)

* HSOPSC = Hospital Survey on Patient Culture; ^†^p-valor

## Discussion

Nursing is an important professional group responsible for the care of hospitalized patients during the COVID-19 pandemic. Inside the nursing team, we highlight the preoccupation with the patient’s safety, disposal of material resources and labor aspects, such as the PPE use by peers. In the evaluation of professional and patient safety, we observed that the COVID-19 diagnosis was more evident among professionals that did not present doubts about removing the PPE, with a higher time of work in the unit and higher scores in the domain “Overall perceptions of patient safety” from HSOPSC. A systematic review with a meta-analysis carried out with 72 studies evidenced that hand disinfection and the use of PPE with individual and collective protection were associated with the reduction of COVID-19 infection^12^.

Research demonstrates that higher exposure to labor risks is due to prolonged contact time with infected patients that need frequent interventions^13^. The findings show that the working time in the unit was associated with COVID-19 infection and most professionals (89.77%) worked between 20 to 39 weeks hours only in one hospital (62.52%). The pandemic demanded a great volume of professionals as a working force, aiming to provide the challenges of the overload of highly complex care activities directed to critical patient care and in extreme vulnerability situations^14^. A cross-sectional study compared the nurses perception which worked in units of COVID-19 and non-COVID-19 about patient security and care quality showed that the professionals who assisted patients with COVID-19 diagnosis presented a significant increase in extra hours (p=0.006), higher distancing due to infection (p<0.001) and significant worsening in the care quality (85.7% versus 98.3%, p = 0.04), classifying the patient safety in the COVID-19 units as significantly lower (76.7% *versus* 94.7%, p = 0.016)^15^.

Nursing professionals experienced, during the beginning of the COVID-19 pandemic, a continuous necessity to consume better available evidence to respond to constant questions, reorganize assistance flows and continuing education. In this research, we observe statistically significant differences between HSOPSC domains that evaluated the patient safety culture and the conduction of training for COVID-19, besides the COVID-19 infections and doubts about removing the PPE and secure flow of patients. 

Given the new challenges, a study carried out in Pakistan with nursing professionals carried out educational interventions and learning through *WhatsApp*, and the results showed that the group participants reported significant improvement in the learning for “infections prevention and control”, “knowledge COVID-19”, as well as “leadership and communication”^16^. Moreover, in this same study, we verified that nursing professionals need longer periods of training and support for continuous learning and not only training restricted to hours or a few days^16^. 

An investigation carried out with 2,707 professionals from 60 countries highlighted that the excessive training demanded in care during the pandemic influenced professional exhaustion, besides the exposure during patient care to COVID-19 and life prioritization; however, the use of PPE positively contributed to minimizing the burnout symptoms^17^. It is important to highlight that the institutional psychological support to the collaborators during the stress confrontation and emotional support during the pandemic were associated with better teamwork, a safe work environment, job satisfaction and job conditions and stress recognition^7^. 

During the COVID-19 pandemic, the managers fulfilled a fundamental role in the infection reduction of individuals in the health institutions, besides being involved in patient care to guarantee the safety and the assistance dynamics based on the best care practices. Many pieces of training were carried out, mainly due to the demand of new hiring, especially professionals without previous experience with patients of high complexity, such as those infected with the coronavirus^3^ ^,^ ^18^ ^-^ ^19^. The management during the peak of service and the necessity of allocating resources to guarantee the hope and well-being of the health professional are essential attributes to balance the patients’ demands in the ICU and the maintenance of the worker’s health status^3^ ^,^ ^18^ ^-^ ^19^. 

Nursing management fulfilled a relevant role in the COVID-19 pandemic; however, in this period, it was possible to evidence vulnerabilities of professionals that provided assistance, such as depression, anxiety, stress and insomnia. The mental health decline of nursing professionals made health institutions implement support measures for them, besides positively enhancing the capacity for confrontation in the prevention and treatment of COVID-19^8^ ^,^ ^20^ ^-^ ^24^. 

The pandemic brought grave consequences that will mark the teams involved in the reception and care of patients with COVID-19. A study carried out intending to respond to questions about the shortage of resources and changes in end-of-life care, due to limited resources and burnout in Brazil, highlighted that the gravity of the COVID-19 pandemic concerning health professionals may be related to the “critical shortage of resources, disparities in the availability of resources among regions with different socioeconomic status”, decision-making for the limitation of investments in treatment and foundation in resources, unleashing the burnout syndrome among health collaborators that may negatively impact the care quality^18^. Observations point to a high psychological burden on health care in ICU during the pandemic with a growth rate of symptoms such as anxiety and mental and physical exhaustion^23^ ^-^ ^24^.

This study contributed to reinforcing the importance of teamwork, mainly about the relevance of active participation of management in the conduction of care through the establishment of safe flows for professionals and patients. Besides that, we evidenced how the decision-making of managers directly impacted the perception of a safe healthcare environment. Furthermore, we hope that the instrument “Professional nursing and patient safety” may contribute to the conduction of future studies aiming at the evaluation of occupational safety in the care of health services. 

Although several investigations have evidenced that patient safety during the COVID-19 pandemic is important, this study evaluates several safety dimensions of the professional and the patient in front of the COVID-19 pandemic in two teaching hospitals that have differences regarding the sociodemographic characteristics, year of foundation and organizational structure. 

The main limitation of this research was evidenced by the low percentage of answers from nursing professionals to the questionnaire. It was necessary to visit the hospital units several times to obtain answers. We collected the data during the first wave of COVID-19 when the epidemiological scenario and the treating possibilities were very unsure and there was no prospect for vaccination, which may have affected the evaluation of nursing professionals about the patient safety culture. However, the study data evidenced the involvement of professionals in the face of their safety and patients with COVID-19. We highlight that the obtained results compared to current scenarios, with better control of the disease, safety in the use of PPE and population vaccination, indicate a decrease in signs and symptoms associated with the gravity of the infection by COVID-19; however, the adopted safety measures during the pandemic brought more safety to the management of the hospitalized patient due to the constant updating of safety measures and institutional training.

## Conclusion

A higher professional experience time of the nursing professional was associated with non-infection by COVID-19. The perception of the safety culture of the patient was related to the “supervisor/manager expectations and actions promoting patient safety”, “organizational learning (continuous improvement)”, “management support for the patient safety” and “feedback and communication about error” were associated with the accomplishment of training directed to the care of suspected/infected by COVID-19.

Strategies that favored the stimulus to the patient safety culture, such as the continuous training of the nursing professional and the enhancement of the working process, were essential for safer flow implementation to the professionals and patients and to improve the occupational safety perception.
